# Supplementary material for: Characterizing the heterogeneity of neurodegenerative diseases through EEG normative modeling
Source: NPJ Parkinsons Dis. 2025 May 8;11:117. doi: 10.1038/s41531-025-00957-6 (PMC12062460; doi:10.1038/s41531-025-00957-6)
Supplement: Supplementary file 1 — SUPPLEMENTAL MATERIAL [file 41531_2025_957_MOESM1_ESM.docx]

Supplementary Materials

**Characterizing the heterogeneity of neurodegenerative diseases through EEG and normative modeling**

**Supplementary Data 1 | Demographic and EEG system details of the control and clinical cohorts used in this study across 14 sites**^1–22^**.**

Supplementary_Data_S1 (Excel File)

**Supplementary Data 2 | Channel indices mapping between the reference EEG system (Nihon Kohden EEG 2100, 19 channels) and the other EEG systems used in this study across 14 sites.**

Supplementary_Data_S2 (Excel File)

## **Exploratory Data Analysis**

We performed a Mann-Whitney U test to check for any significant differences in the EEG spectral power and functional connectivity distribution between male and female participants within each group. The effect sizes of these differences were quantified using Cohen’s d.


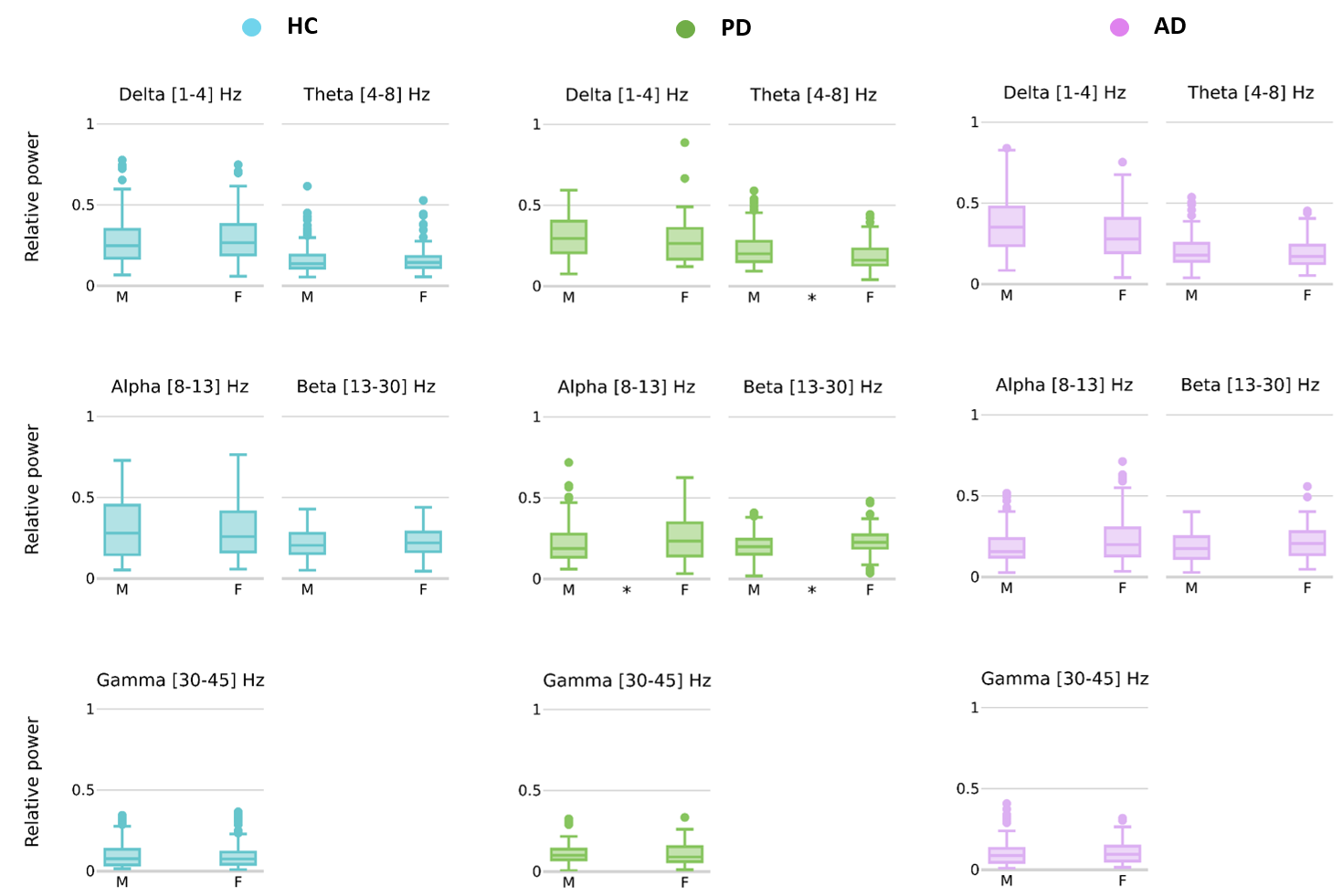


**Supplementary Figure 1 | Sex differences in the averaged relative power of EEG frequency bands in Healthy Controls (HC), Parkinson’s Disease (PD) and Alzheimer’s Disease (AD) groups.** (*) denote statistically significant differences between sexes within each frequency band.

**Supplementary Table 1 | Sex differences in the averaged relative power of EEG frequency bands: *P-value (Cohen’s d).***

|  | **Delta [1-4] Hz** | **Theta [4-8] Hz** | **Alpha [8-13] Hz** | **Beta [13-30] Hz** | **Gamma [30-45] Hz** |
| --- | --- | --- | --- | --- | --- |
| **HC** | 0.06 (-0.15) | 0.75 (0.04) | 0.63 (0.07) | 0.16 (-0.11) | 0.39 (0.22) |
| **PD** | 0.09 (0.18) | 0.00 (0.46) | 0.02 (-0.35) | 0.01 (-0.33) | 0.70 (0.03) |
| **AD** | 0.06 (0.29) | 0.42 (0.18) | 0.06 (-0.32) | 0.11 (-0.25) | 0.46 (0.04) |


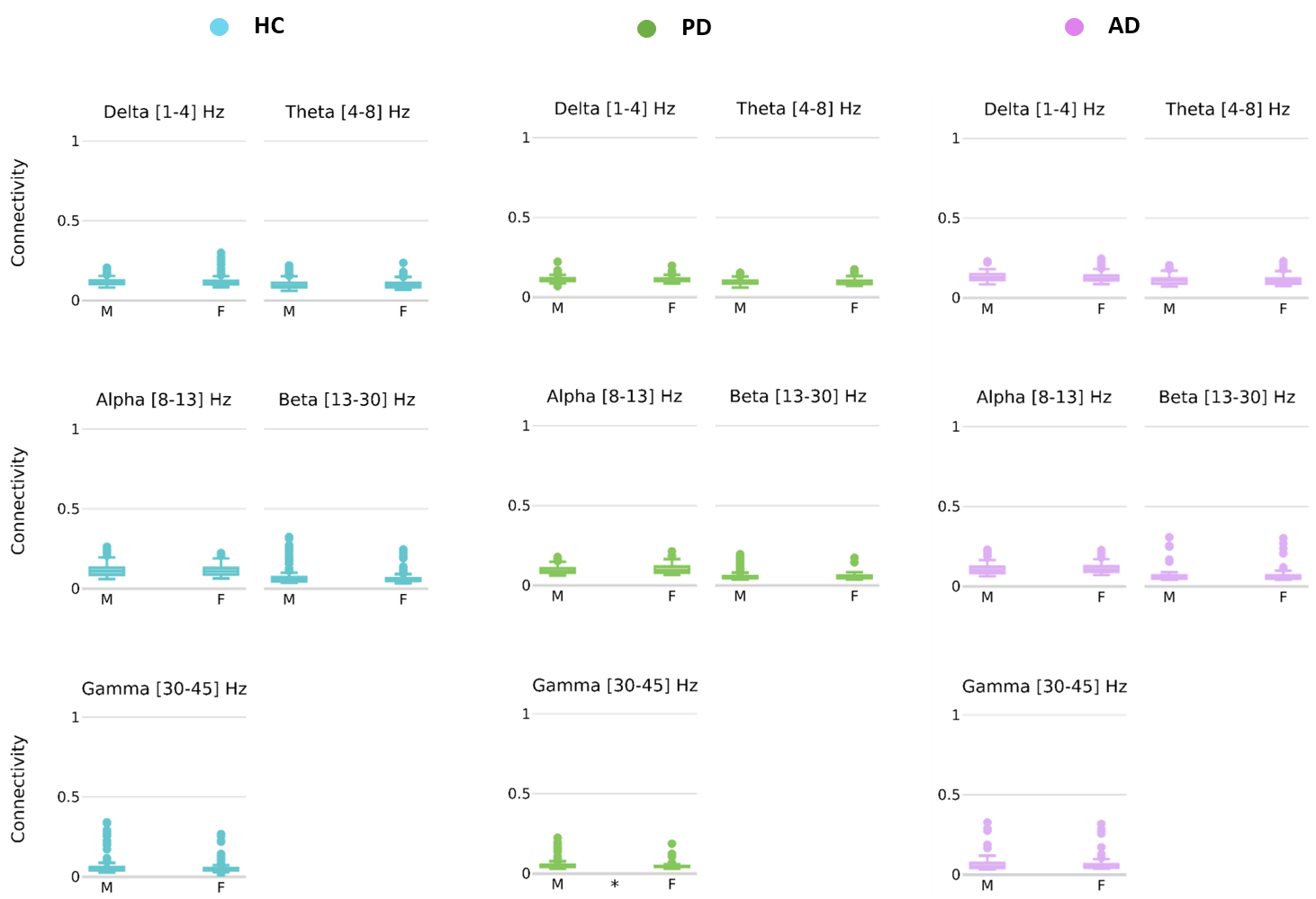


**Supplementary Figure 2 | Sex differences in the averaged functional connectivity of EEG frequency bands in Healthy Controls (HC), Parkinson’s Disease (PD) and Alzheimer’s Disease (AD) groups.** (*) denote statistically significant differences between sexes within each frequency band.

**Supplementary Table 2 | Sex differences in the averaged functional connectivity of EEG frequency bands: *P-value (Cohen’s d).***

|  | **Delta [1-4] Hz** | **Theta [4-8] Hz** | **Alpha [8-13] Hz** | **Beta [13-30] Hz** | **Gamma [30-45] Hz** |
| --- | --- | --- | --- | --- | --- |
| **HC** | 0.27 (-0.05) | 0.81 (0.05) | 0.84 (0.06) | 0.37 (0.25) | 0.11 (0.26) |
| **PD** | 0.22 (0.12) | 0.45 (-0.06) | 0.11 (-0.28) | 0.61 (0.08) | 0.00 (0.28) |
| **AD** | 0.07 (0.17) | 0.78 (-0.01) | 0.27 (-0.12) | 0.96 (0.11) | 0.81 (0.11) |

**Supplementary Table 3 | Sex and age differences in the groups and datasets*.***

| **Comparisons** | **Statistical Test** | **p-value** |
| --- | --- | --- |
| Sex across groups | Chi-square | 7.73E-09 * |
| Sex across datasets | Chi-square | 0.00087828 * |
| Age across groups | Kruskal-Wallis | 7.04E-38 * |
| Age across datasets | Kruskal-Wallis | 6.66E-50 * |

## **Normative Modeling**

### **Model distribution and covariates**

**Supplementary Table 4 | Model equations and family distribution for spectral features models across all frequency bands**

| **Frequency band** | **Delta** | **Theta** | **Alpha** | **Beta** | **Gamma** |
| --- | --- | --- | --- | --- | --- |
| **mu** | y ~ fp (age, 1) | y ~ fp (age, 1) | y ~ fp (age, 1) | y ~ fp (age, 1) | y ~ fp (age, 1) |
| **sigma** | ~ 1 | ~ 1 | ~ 1 | ~ 1 | ~ 1 |
| **Distribution Family** | SEP4 | GG | SEP4 | GG | GG |

**Supplementary Table 5 | Model equations and family distribution for connectivity features models across all frequency bands**

| **Frequency band** | **Delta** | **Theta** | **Alpha** | **Beta** | **Gamma** |
| --- | --- | --- | --- | --- | --- |
| **mu** | y ~ fp (age, 1) + factor (sex) | y ~ fp (age, 1) | y ~ fp (age, 1) | y ~ fp (age, 1) | y ~ fp (age, 1) |
| **sigma** | ~ fp (age, 1) | ~ fp (age, 1) | ~ fp (age, 1) | ~ fp (age, 1) | ~ fp (age, 1) |
| **nu** | ~ 1 | ~ 1 | ~ 1 | ~ 1 | ~ 1 |
| **Distribution Family** | ST3 | SEP3 | SEP4 | ST3 | ST1 |

### **Model performance**


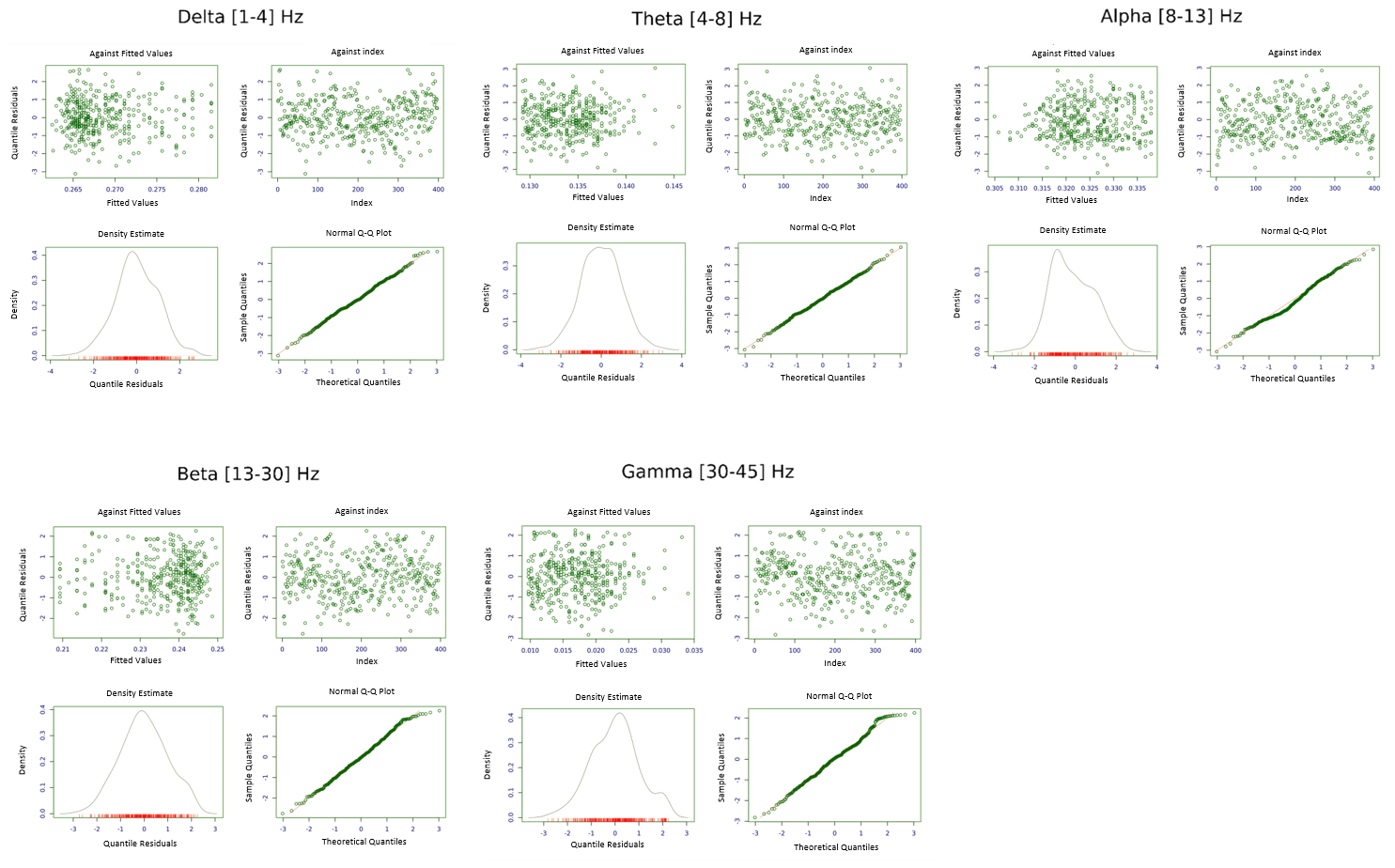


**Supplementary Figure 3 | Diagnostic residual plots for assessing spectral model fit:** residuals *vs* fitted values, index, density estimate, and normal Q-Q plot.


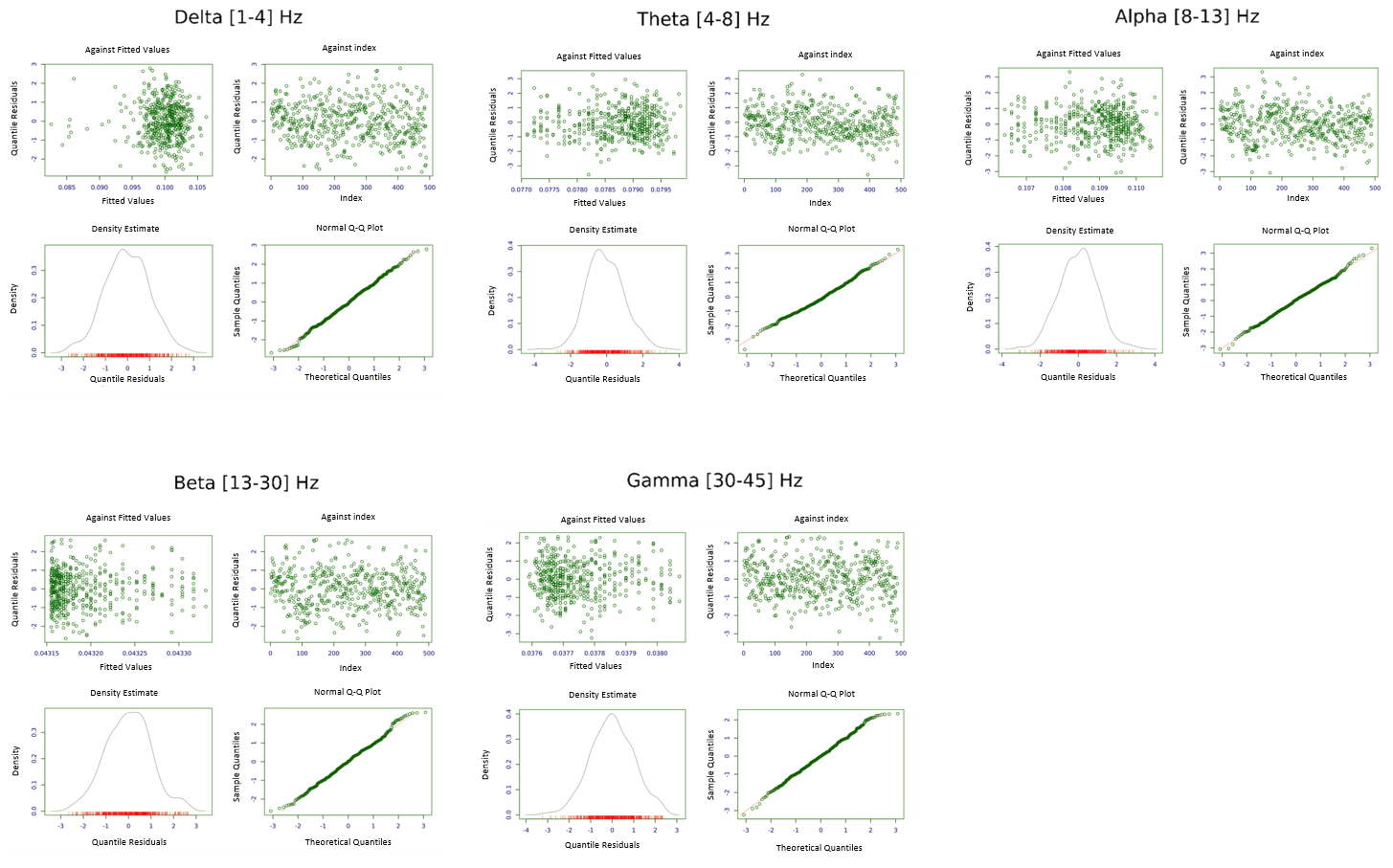


**Supplementary Figure 4 | Diagnostic residual plots for assessing connectivity model fit:** residuals *vs* fitted values, index, density estimate, and normal Q-Q plot.

### **Model Sensitivity**

**
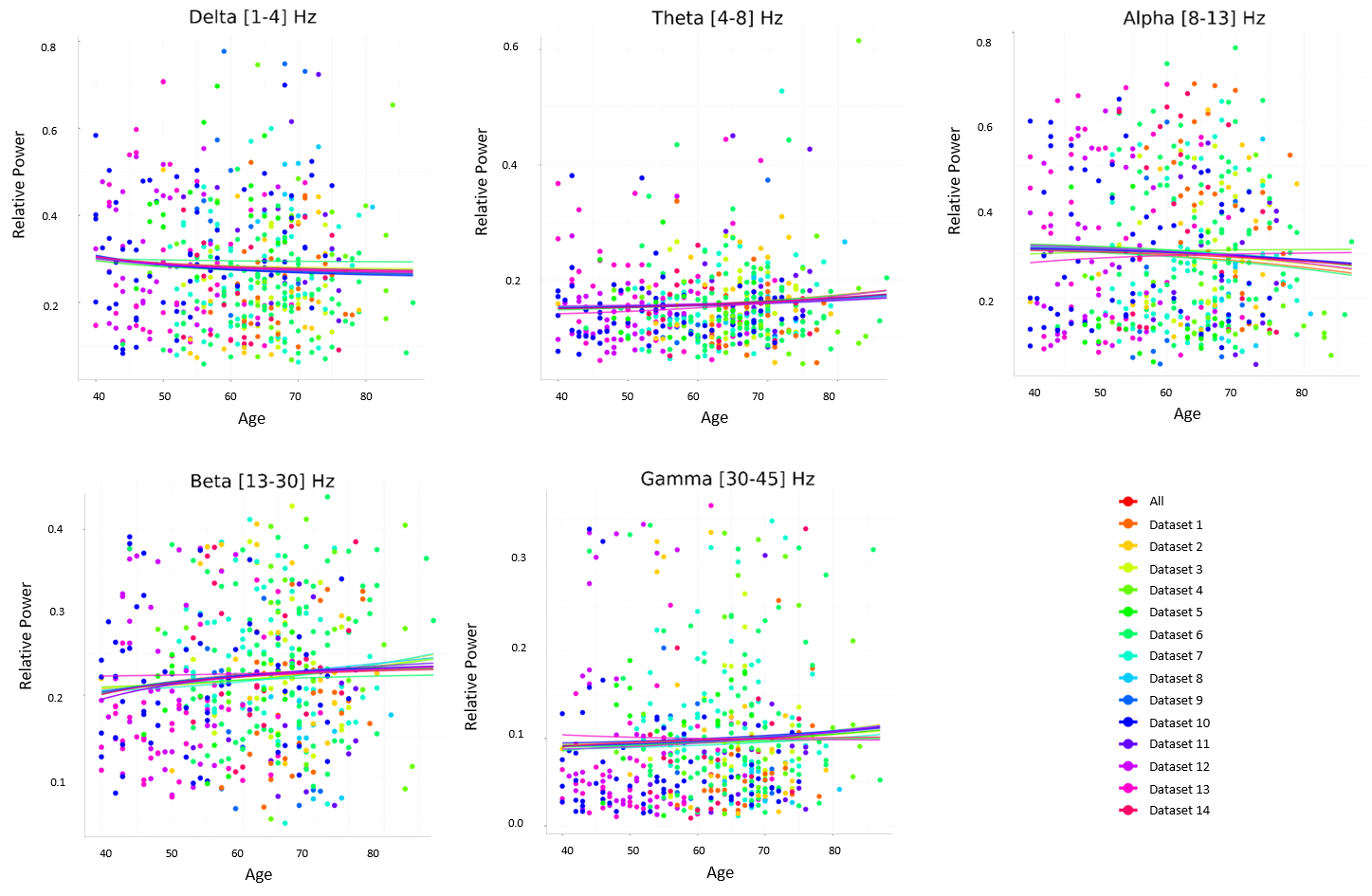
**

**Supplementary Figure 5 | Leave One Study Out (LOSO) analysis for assessing spectral model sensitivity on datasets:** normative model trajectories across age excluding one dataset per iteration, compared to full dataset trajectories (red) across all frequency bands.


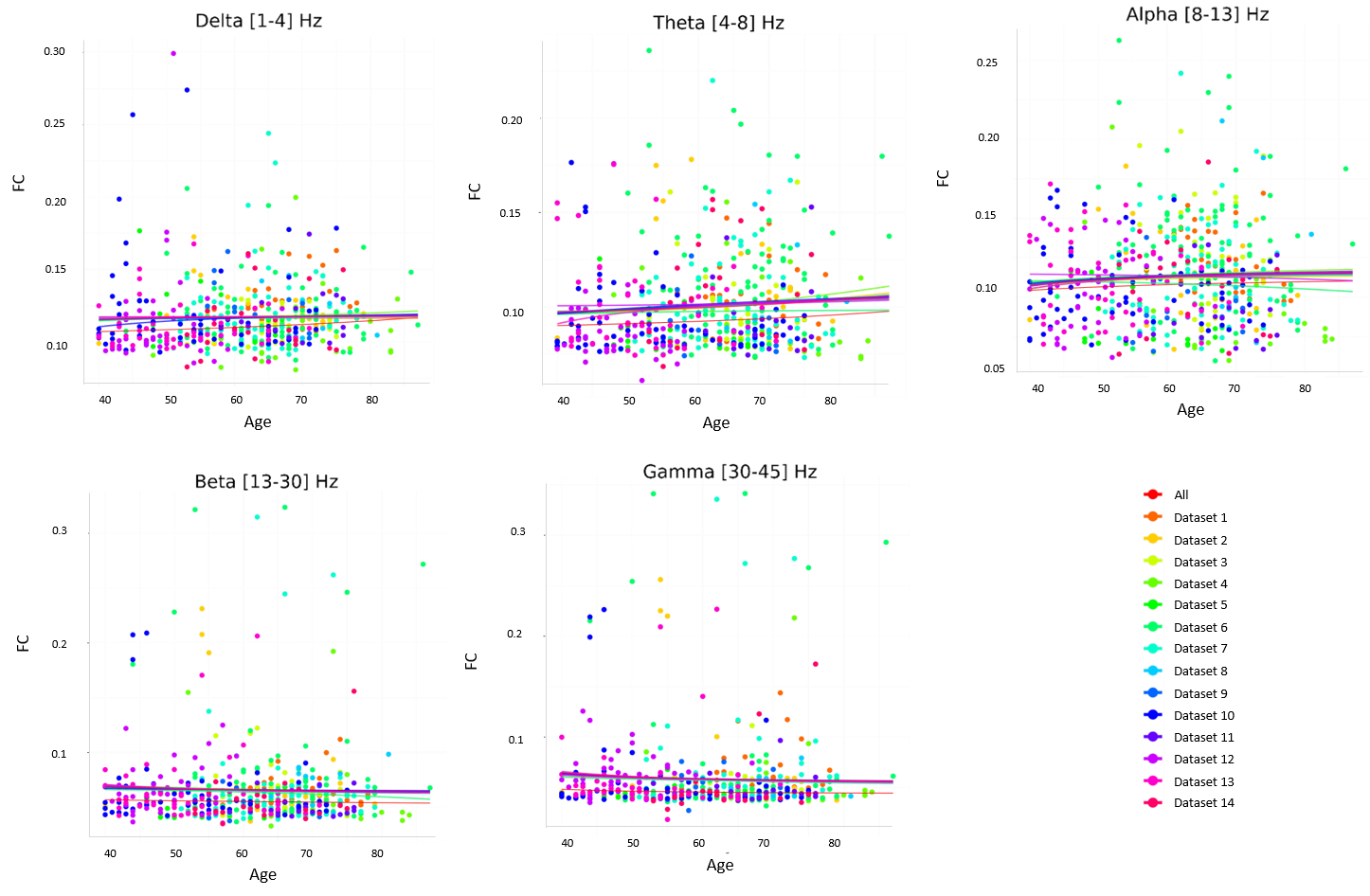


**Supplementary Figure 6 | Leave One Study Out (LOSO) analysis for assessing FC model sensitivity on datasets:** normative model trajectories across age excluding one dataset per iteration, compared to full dataset trajectories (red) across all frequency bands.


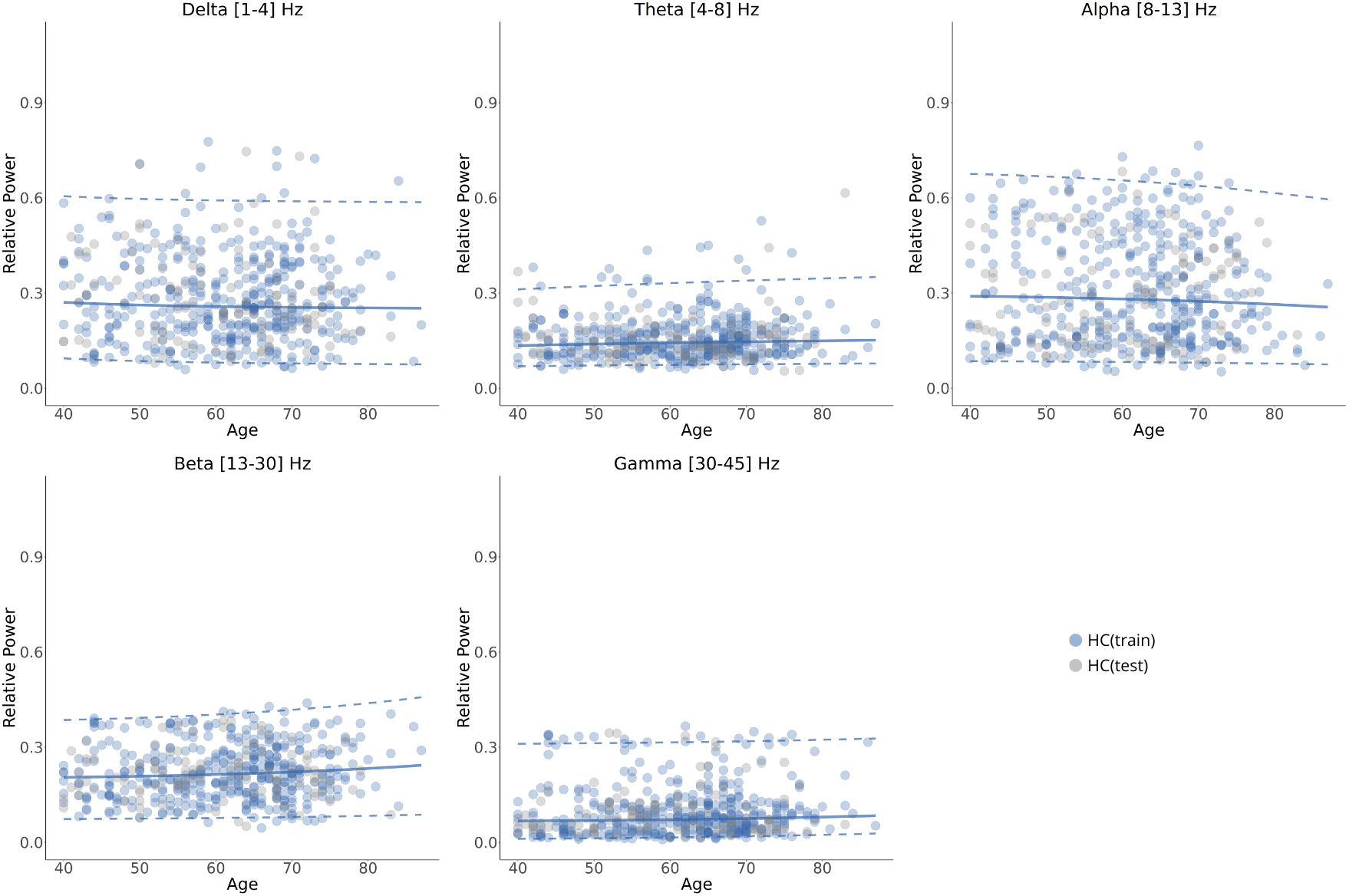


**Supplementary Figure 7 | Normative model aging trajectories of spectral features across age within each frequency band along with patients data.** The median (50th percentile) is depicted with a solid blue line, while the 5th and 95th percentiles are indicated by dotted blue lines. The solid black line represents the mean parameter of the GAMLSS fit model. Relative power averaged over channels are represented by scatter points for healthy and patient groups (HC(train), HC(test).


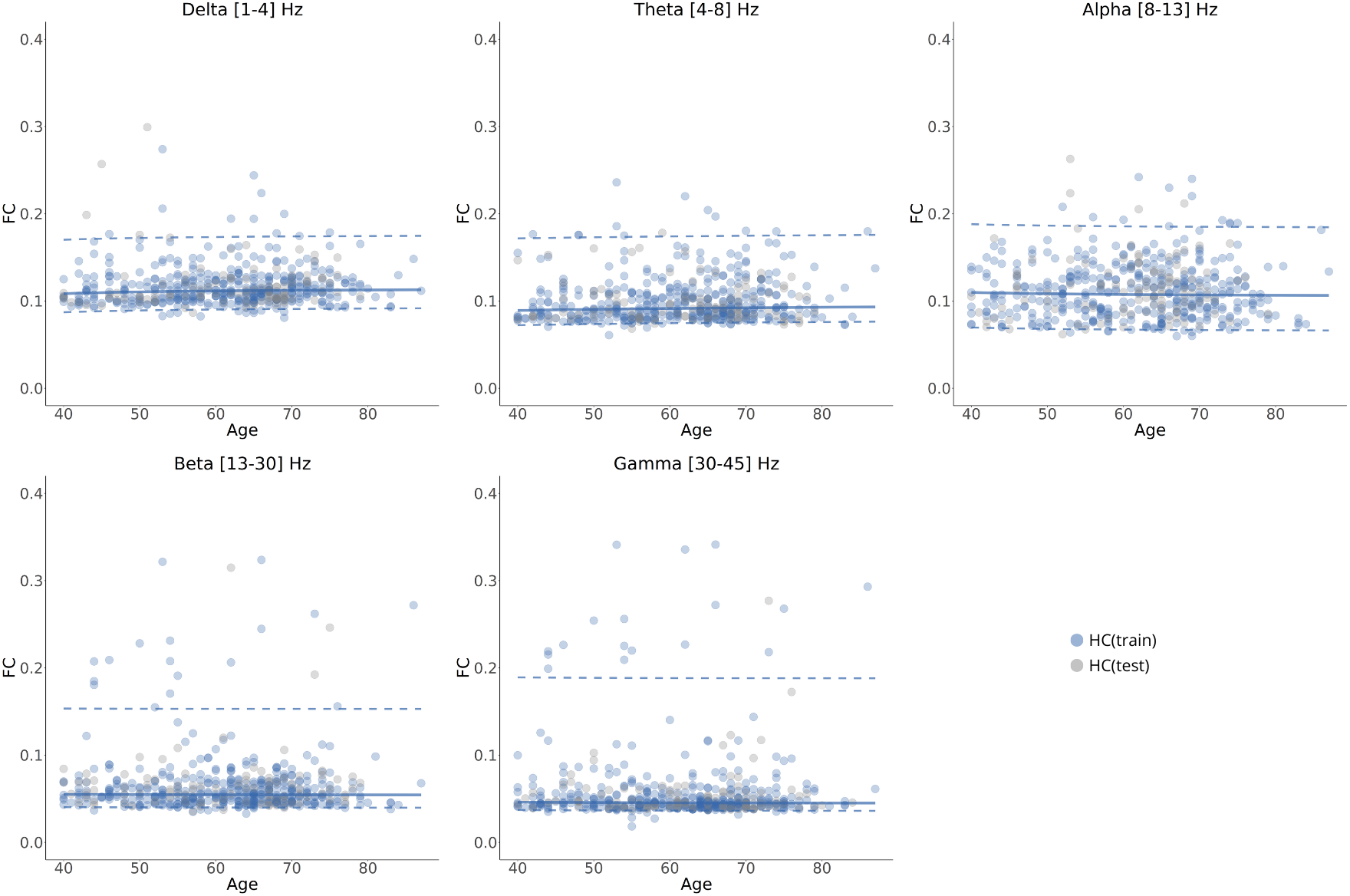


**Supplementary Figure 8 | Normative model aging trajectories of functional connectivity features across age within each frequency band along with patients data.** The median (50th percentile) is depicted with a solid blue line, while the 5th and 95th percentiles are indicated by dotted blue lines. The solid black line represents the mean parameter of the GAMLSS fit model. FC values averaged over connections are represented by scatter points for healthy and patient groups (HC(train), HC(test).


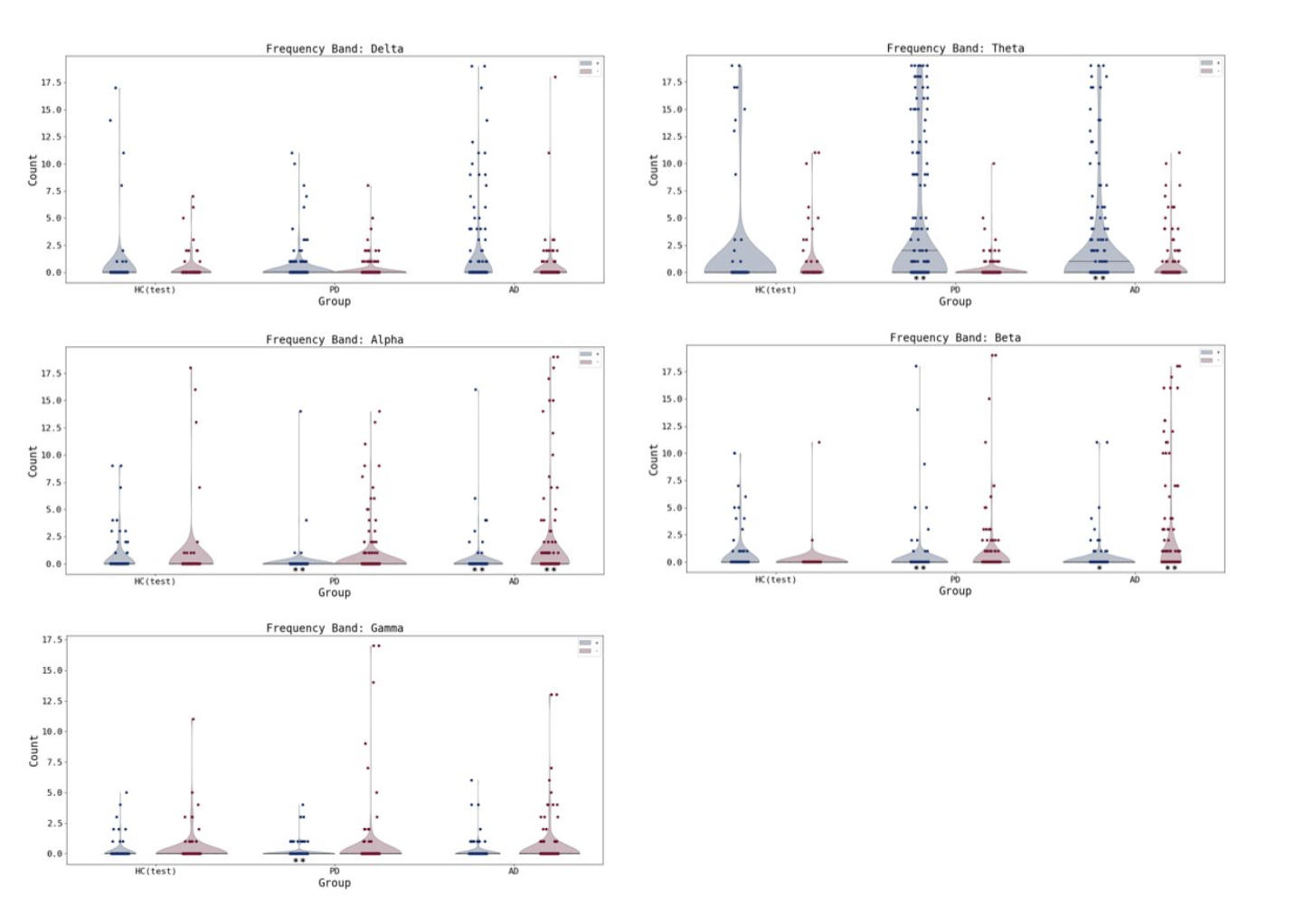


**Supplementary Figure 9 | Distribution of the number of extremely deviated channels per subject across groups within each frequency band.** Blue (Red) violins represent positive (negative) deviations. (*) denotes significant difference between HC and cases *p<0.05*. (**) denotes significant difference between HC and cases *p<0.01*.


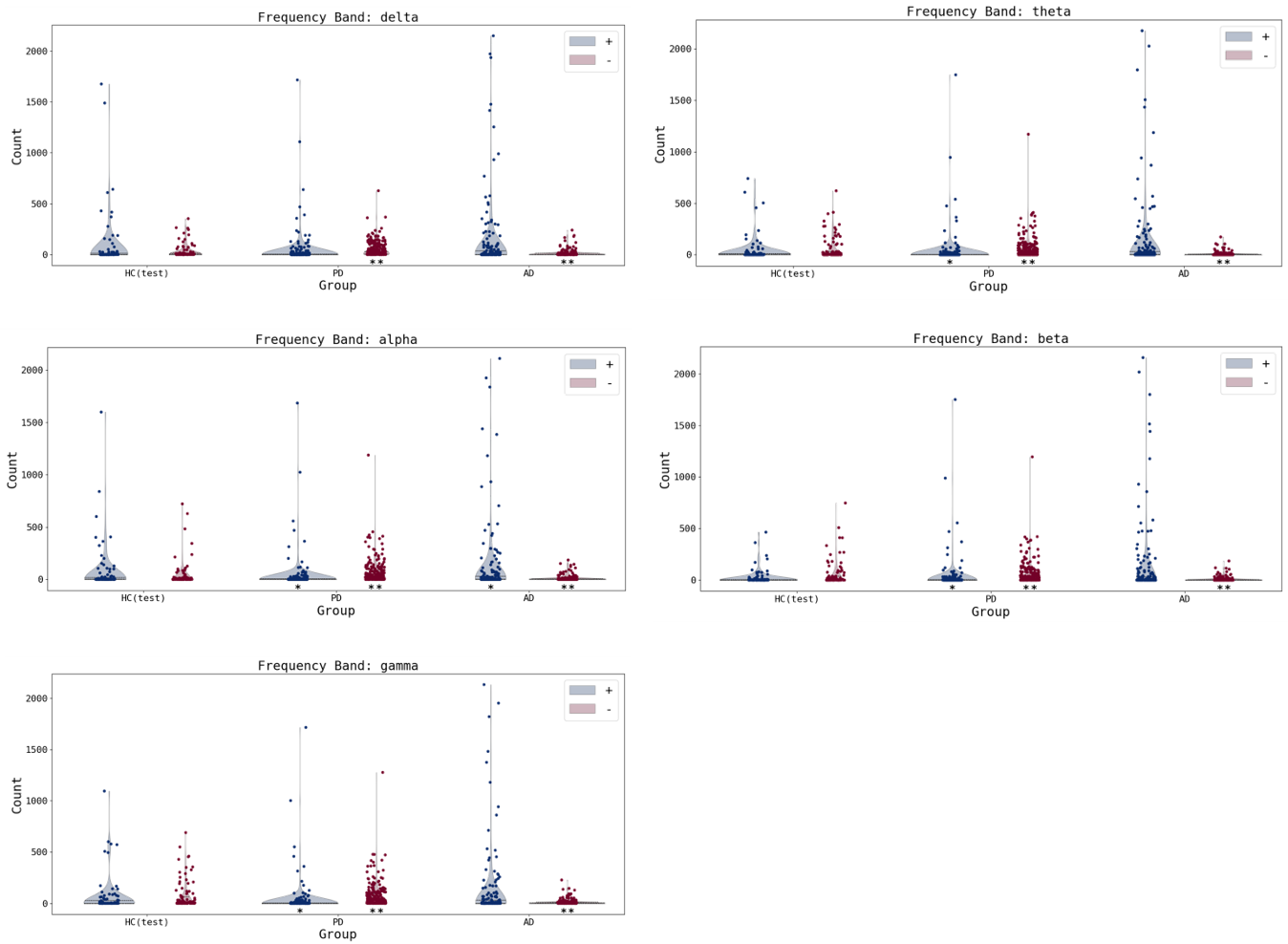


**Supplementary Figure 10 | Distribution of the number of extremely deviated connections per subject across groups within each frequency band.** Blue (Red) violins represent positive (negative) deviations. (*) denotes significant difference between HC and cases *p<0.05*. (**) denotes significant difference between HC and cases *p<0.01*.

**Supplementary Table 6 | Percentage of subjects exhibiting at least one extremely deviant channel and the median number of extreme deviations across groups within each frequency band.**

| **Group** | **% at least one positive deviation** | **Median [range]**  **positive deviation** | **% at least one negative deviation** | **Median [range]**  **negative deviation** |
| --- | --- | --- | --- | --- |
|  |  | **Delta** |  |  |
| HC(test) | 8.08 | 0.0 [0-17] | 10.10 | 0.0 [0-7] |
| PD | 13.14 | 0.0 [0-11] | 8.47 | 0.0 [0-8] |
| AD | 17.77 | 0.0 [0-19] | 9.14 | 0.0 [0-18] |
|  |  | **Theta** |  |  |
| HC(test) | 13.27 | 0.0 [0-19] | 14.29 | 0.0 [0-11] |
| PD | 31.36 | 0.0 [0-19] | 7.63 | 0.0 [0-10] |
| AD | 27.41 | 0.0 [0-19] | 13.71 | 0.0 [0-11] |
|  |  | **Alpha** |  |  |
| HC(test) | 15.15 | 0.0 [0-9] | 9.09 | 0.0 [0-18] |
| PD | 1.69 | 0.0 [0-14] | 11.86 | 0.0 [0-14] |
| AD | 4.57 | 0.0 [0-16] | 18.78 | 0.0 [0-19] |
|  |  | **Beta** |  |  |
| HC(test) | 16.16 | 0.0 [0-10] | 2.02 | 0.0 [0-11] |
| PD | 4.66 | 0.0 [0-18] | 12.71 | 0.0 [0-19] |
| AD | 6.60 | 0.0 [0-11] | 23.35 | 0.0 [0-18] |
|  |  | **Gamma** |  |  |
| HC(test) | 9.00 | 0.0 [0-18] | 12.00 | 0.0 [0-10] |
| PD | 5.51 | 0.0 [0-6] | 6.36 | 0.0 [0-18] |
| AD | 7.61 | 0.0 [0-9] | 12.18 | 0.0 [0-16] |

**
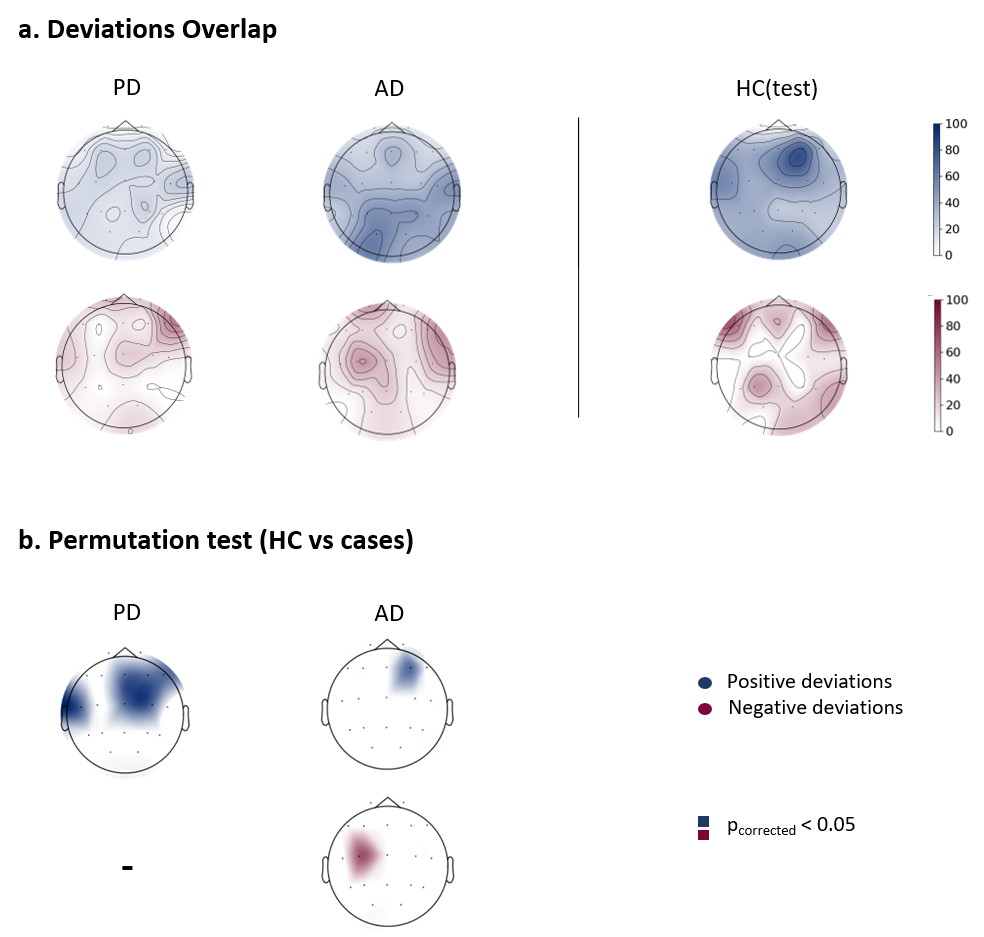
**

**Supplementary Figure 11 | Spectral features maps in the delta band.** a) Overlap maps of deviation scores for clinical groups and the held-out healthy control group (HC(test)), illustrating areas of common deviation. b) channels showing significant differences between HC(test) and clinical groups, determined through group-based permutation tests (p<0.05, FDR corrected).

**
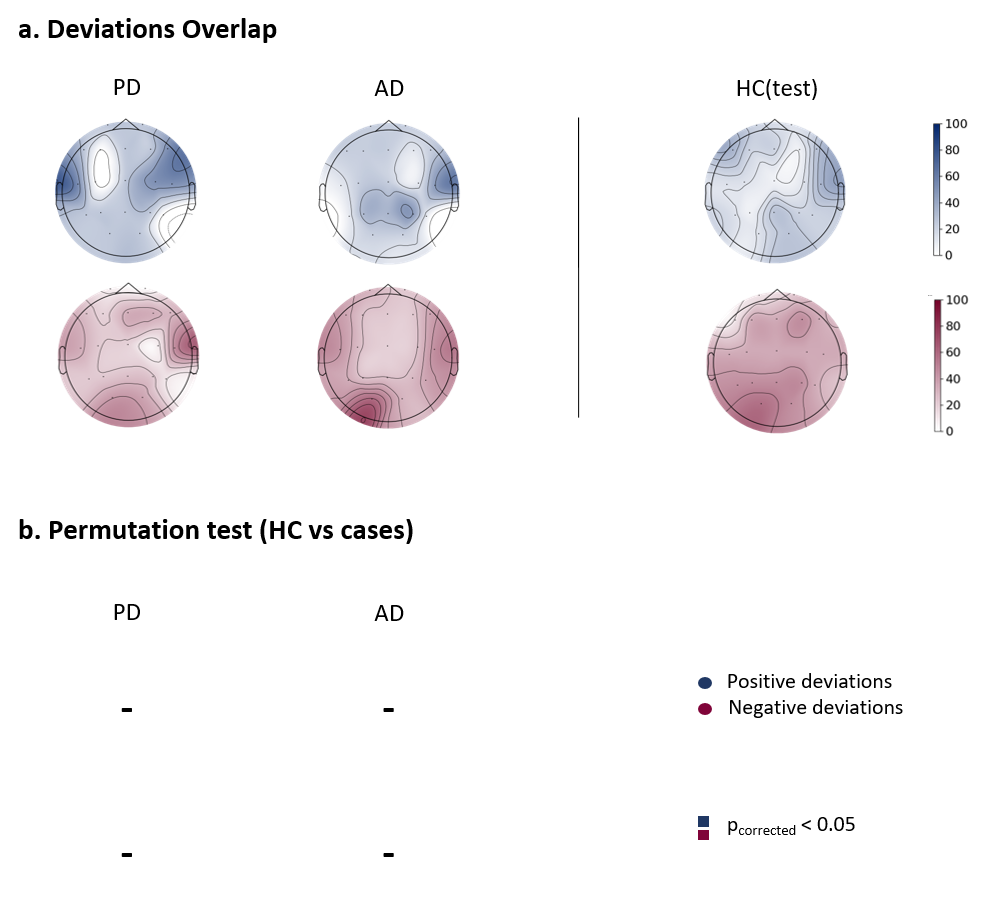
**

**Supplementary Figure 12 | Spectral features maps in the alpha band.** a) Overlap maps of deviation scores for clinical groups and the held-out healthy control group (HC(test)), illustrating areas of common deviation. b) channels showing significant differences between HC(test) and clinical groups, determined through group-based permutation tests (p<0.05, FDR corrected).


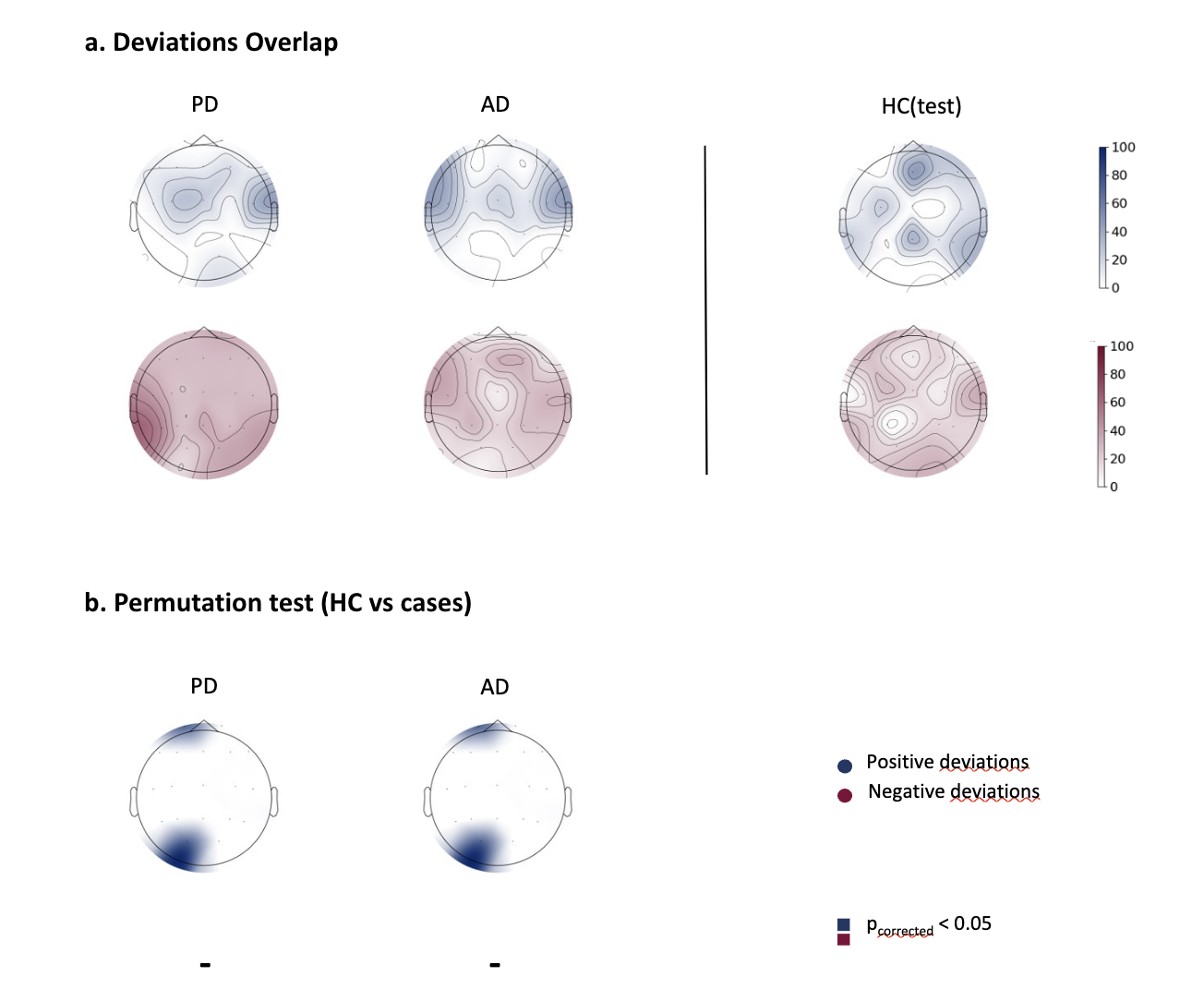


**Supplementary Figure 13 | Spectral features maps in the gamma band.** a) Overlap maps of deviation scores for clinical groups and the held-out healthy control group (HC(test)), illustrating areas of common deviation. b) channels showing significant differences between HC(test) and clinical groups, determined through group-based permutation tests (p<0.05, FDR corrected).

**Supplementary Table 7 | Percentage of subjects exhibiting at least one extremely deviant connection and the median number of extreme deviations across groups within each frequency band.**

| **Group** | **% at least one positive deviation** | **Median [range]**  **positive deviation** | **% at least one negative deviation** | **Median [range]**  **negative deviation** |
| --- | --- | --- | --- | --- |
|  |  | **Delta** |  |  |
| HC(test) | 39.80 | 0.0 [0-1676] | 72.45 | 3.0 [0-353] |
| PD | 40.68 | 0.0 [0-1716] | 86.86 | 26.0 [0-627] |
| AD | 45.69 | 0.0 [0-2150] | 53.81 | 1.0 [0-240] |
|  |  | **Theta** |  |  |
| HC(test) | 42.42 | 0.0 [0-740] | 70.71 | 4.0 [0-621] |
| PD | 35.17 | 0.0 [0-1747] | 83.47 | 17.5 [0-1170] |
| AD | 46.70 | 0.0 [0-2175] | 49.24 | 0.0 [0-173] |
|  |  | **Alpha** |  |  |
| HC(test) | 45.92 | 0.0 [0-1598] | 54.08 | 1.0 [0-721] |
| PD | 38.14 | 0.0 [0-1684] | 83.05 | 19.0 [0-1187] |
| AD | 52.79 | 1.0 [0-2110] | 53.30 | 1.0 [0-185] |
|  |  | **Beta** |  |  |
| HC(test) | 42.27 | 0.0 [0-464] | 62.89 | 2.0 [0-747] |
| PD | 37.71 | 0.0 [0-1750] | 83.05 | 17.0 [0-1194] |
| AD | 46.70 | 0.0 [0-2157] | 49.75 | 0.0 [0-185] |
|  |  | **Gamma** |  |  |
| HC(test) | 42.86 | 0.0 [0-1095] | 73.47 | 7.0 [0-690] |
| PD | 37.29 | 0.0 [0-1716] | 85.59 | 26.0 [0-1277] |
| AD | 44.16 | 0.0 [0-2134] | 54.82 | 1.0 [0-228] |

**
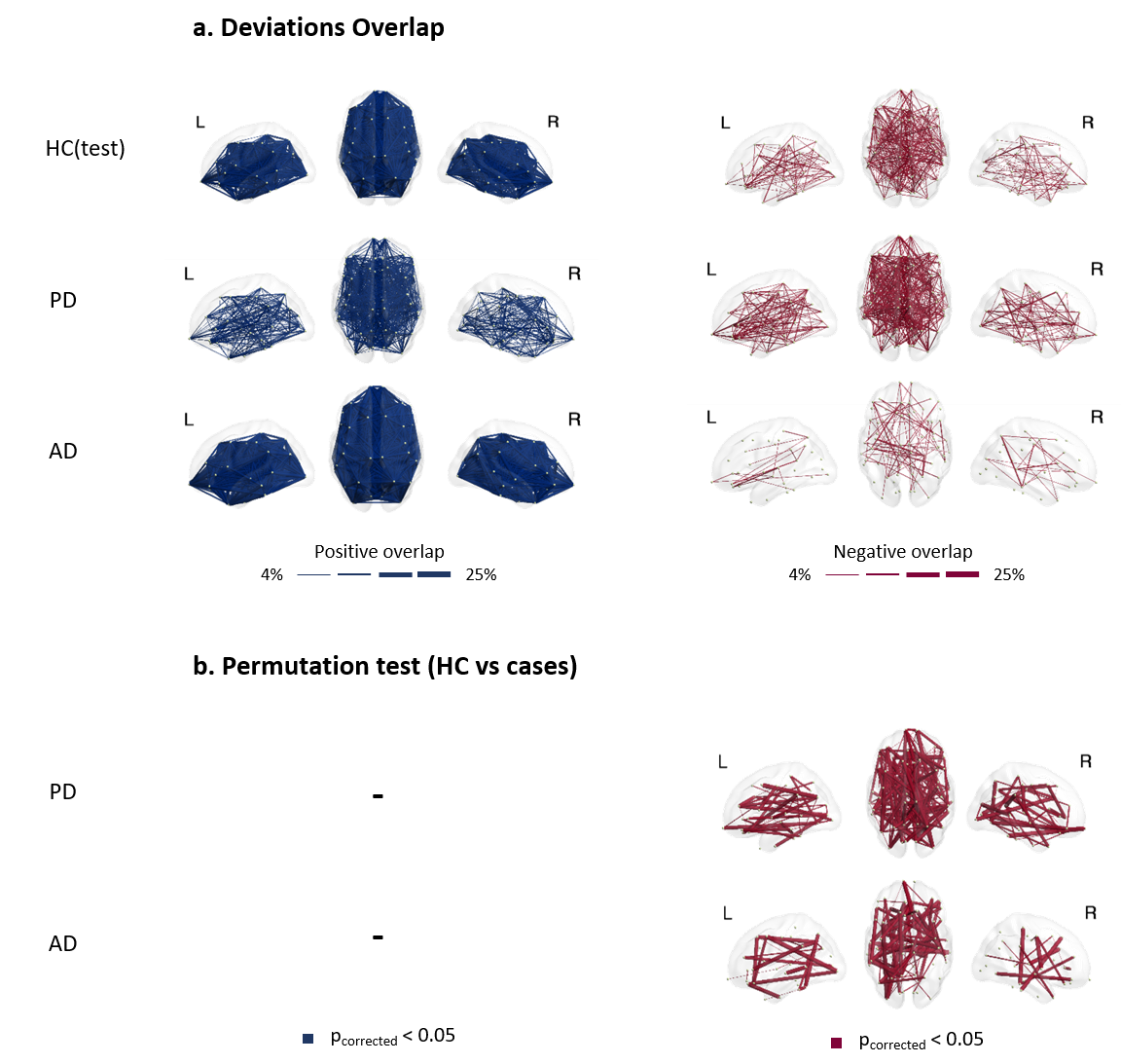
**

**Supplementary Figure 14 | Functional connectivity maps in delta band. a)** Overlap maps of positive and negative deviation scores for clinical groups and the held-out healthy control group (HC(test)), illustrating areas of common deviation among patients (with only the highest 4% overlap values being plotted for visualization purposes). **b)** functional connections showing significant differences between HC(test) and clinical groups, determined through group-based permutation tests (p<0.05, FDR corrected).

**
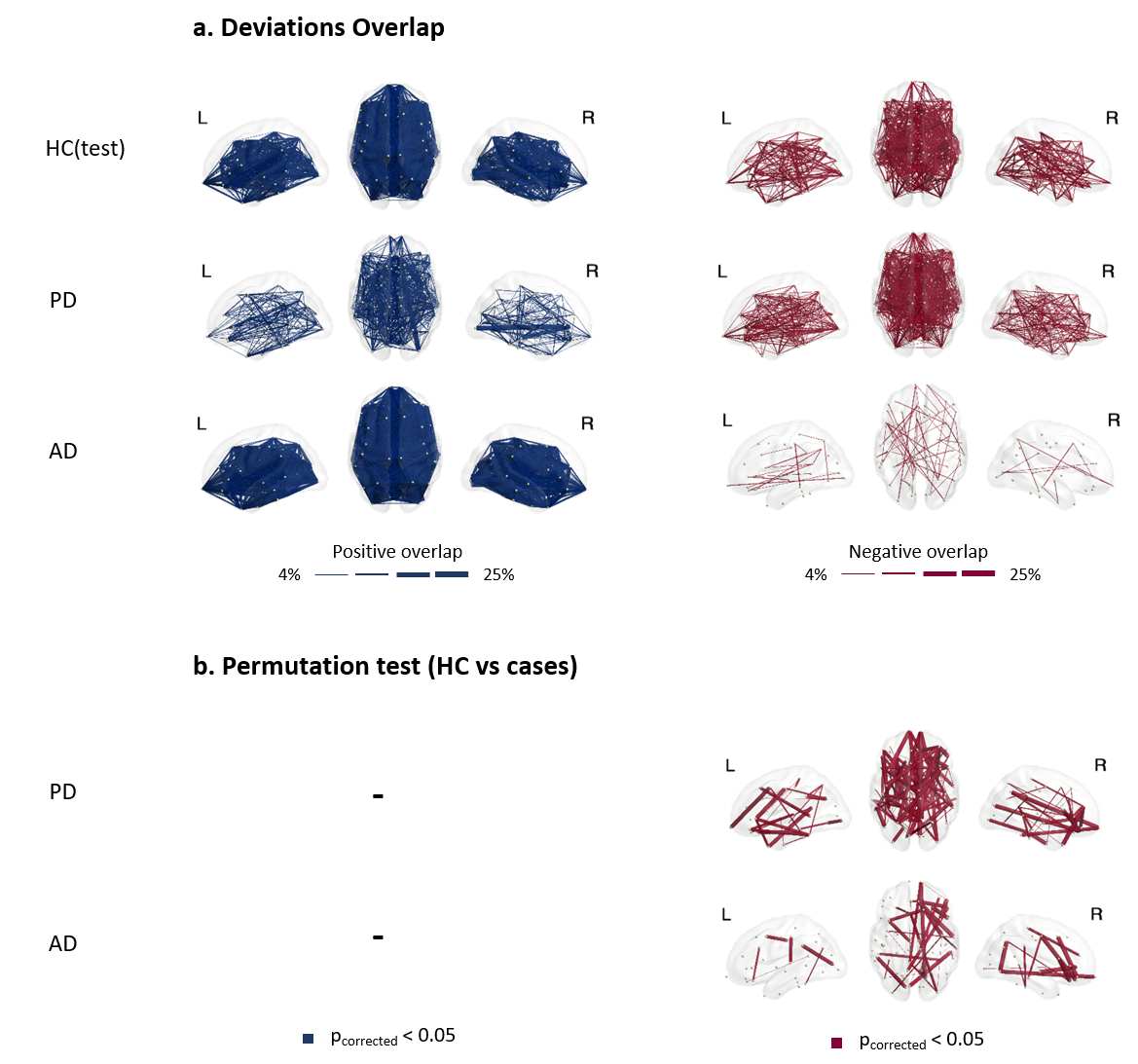
**

**Supplementary Figure 15 | Functional connectivity maps in alpha band. a)** Overlap maps of positive and negative deviation scores for clinical groups and the held-out healthy control group (HC(test)), illustrating areas of common deviation among patients (with only the highest 4% overlap values being plotted for visualization purposes). **b)** functional connections showing significant differences between HC(test) and clinical groups, determined through group-based permutation tests (p<0.05, FDR corrected).

**
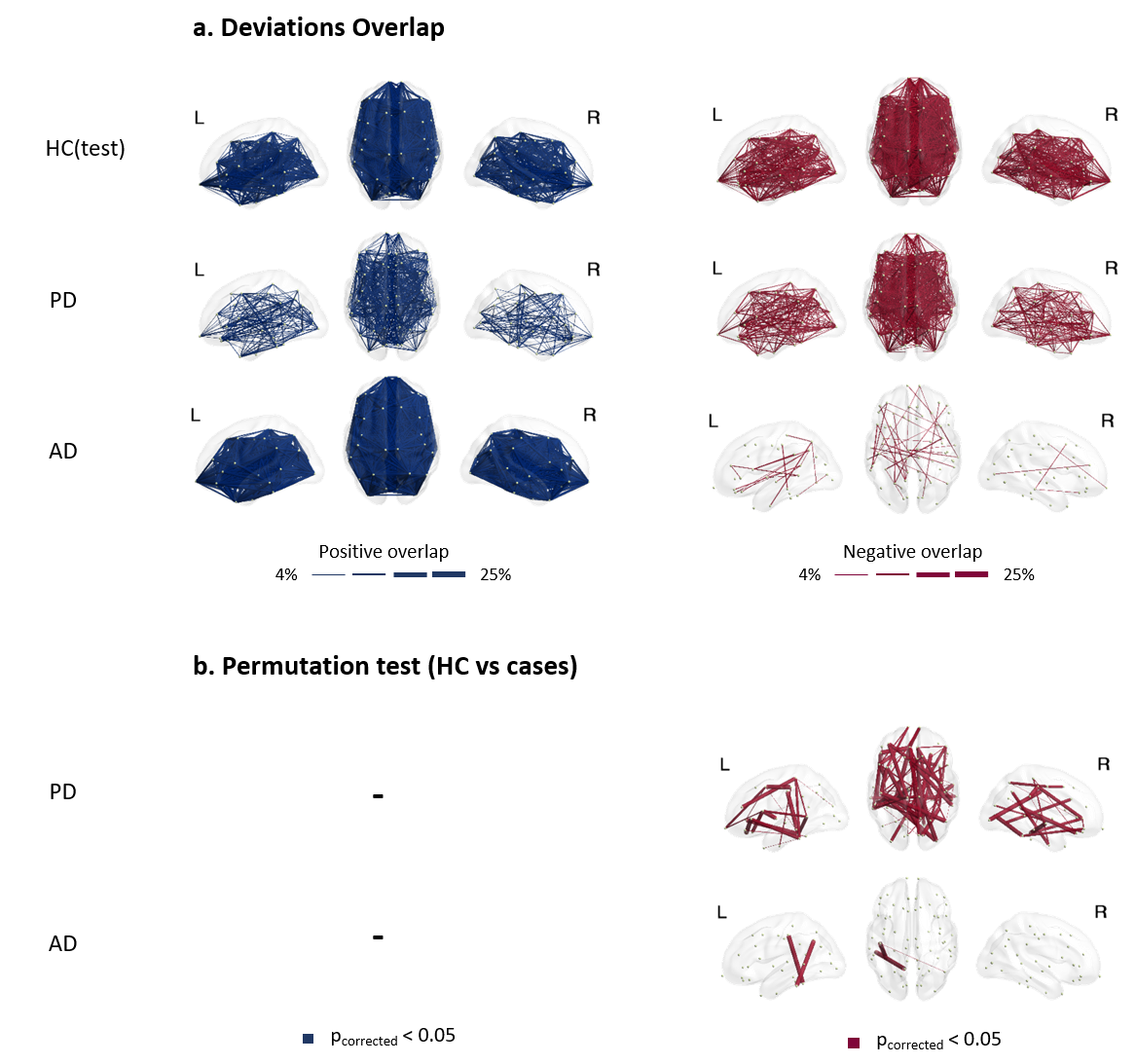
**

**Supplementary Figure 16 | Functional connectivity maps in gamma band. a)** Overlap maps of positive and negative deviation scores for clinical groups and the held-out healthy control group (HC(test)), illustrating areas of common deviation among patients (with only the highest 4% overlap values being plotted for visualization purposes). **b)** Functional connections showing significant differences between HC(test) and clinical groups, determined through group-based permutation tests (p<0.05, FDR corrected).

**Supplementary Table 8 | Distribution of the proportion of the significant connections (after permutation test) in overlap maps across resting state networks for clinical groups in all frequency bands.** Values in red indicate proportions above 35%. *SAN denotes salience, DMN denotes default mode, VIS denotes visual, TEMP denotes temporal, CCN denotes cognitive control, FPN denotes frontoparietal, AUD denotes auditory, SMN denotes somatomotor, and DAN denotes dorsal attention networks.*


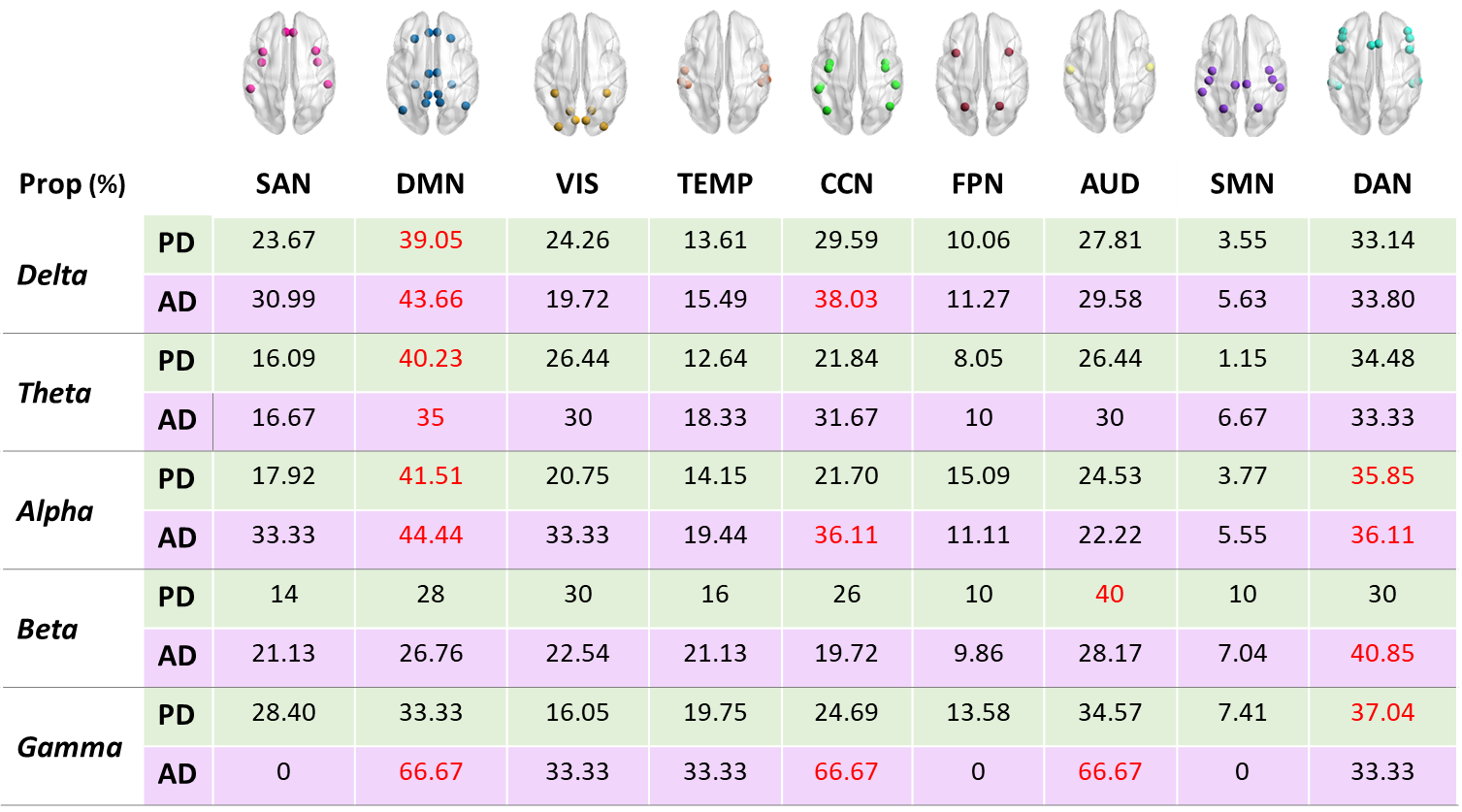


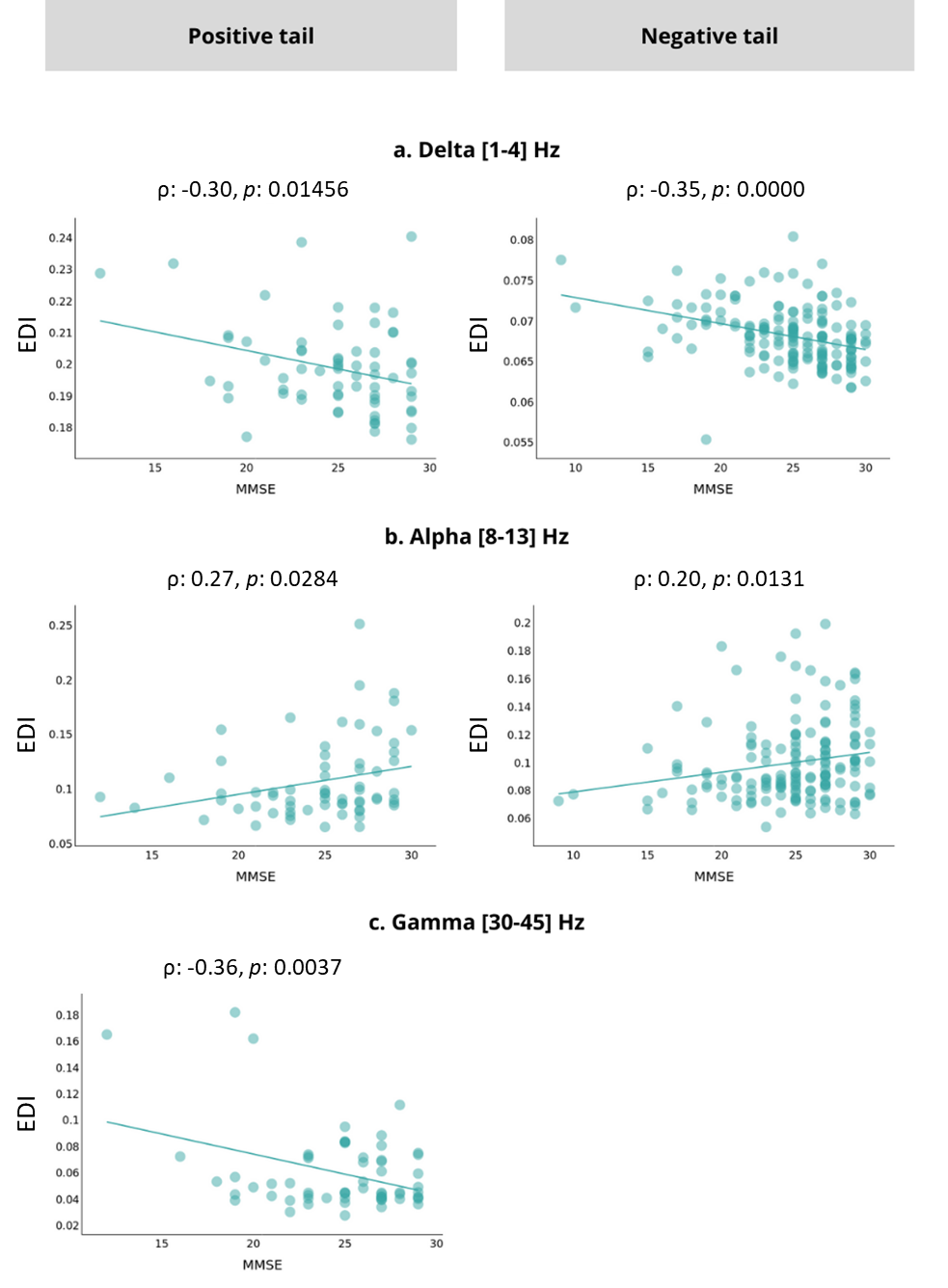


**Supplementary Figure 17 | Significant correlations between PD subjects' scores (EDI) and clinical assessment scores (MMSE) (p-value<0.05) in delta, alpha, and gamma bands respectively. (a, b, and c)** Scores are computed as the averaged FC features over the extremely deviated connections (positive and negative). The corresponding correlation coefficient and p-value are indicated for each case.**
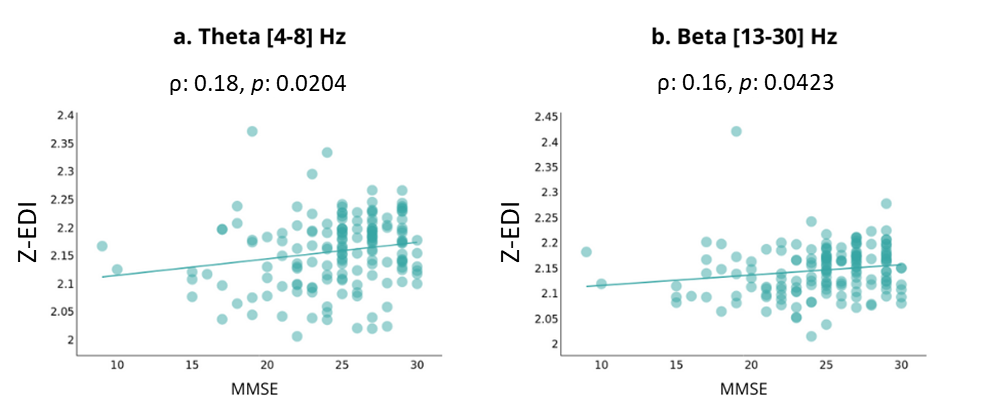
**

**Supplementary Figure 18 | Significant correlation between PD subjects' z-scores (z-EDI) and clinical assessment scores (MMSE) (p-value<0.05) in theta, and beta bands respectively, negative tail. (a, b)** Scores are computed as the averaged z-scores over the extremely deviated connections for FC features. The corresponding correlation coefficient and p-value are indicated for each case.

**Supplementary Table 9 | Distribution of Parkinson’s Disease (PD) and Alzheimer’s Disease (AD) patients by ON- and OFF-medication conditions across datasets.**

|  | **PD** | | **AD** | |
| --- | --- | --- | --- | --- |
|  | **ON-medication** | **OFF-medication** | **ON-medication** | **OFF-medication** |
| **Dataset 1** |  |  | **–** | **–** |
| **Dataset 3** | **68** |  |  |  |
| **Dataset 4** | **13** | **12** |  |  |
| **Dataset 5** | **24** |  |  |  |
| **Dataset 6** |  |  | **–** | **–** |
| **Dataset 7** | **100** |  | **44** |  |
| **Dataset 8** |  |  |  | **10** |
| **Dataset 9** |  |  | **8** | **5** |
| **Dataset 11** | **13** | **7** |  |  |
| **Total** | **218** | **19** | **52** | **15** |

**
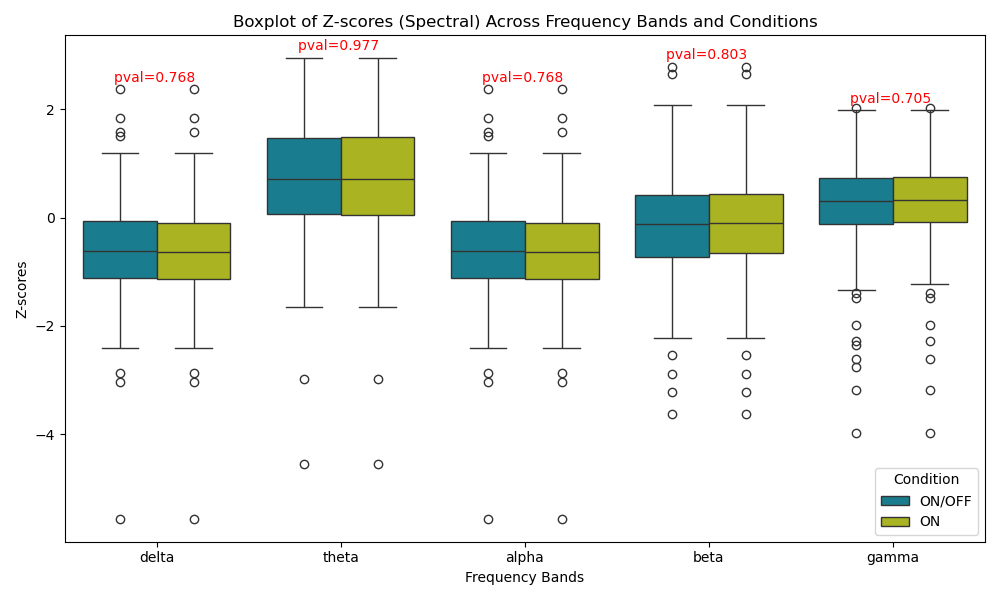
**

**Supplementary Figure 19 | Boxplots of z-score deviations for spectral features across frequency bands, comparing ON/OFF-medication and ON-only medication conditions for PD patients.** Mann-Whitney U test p-values are indicated in red.

**
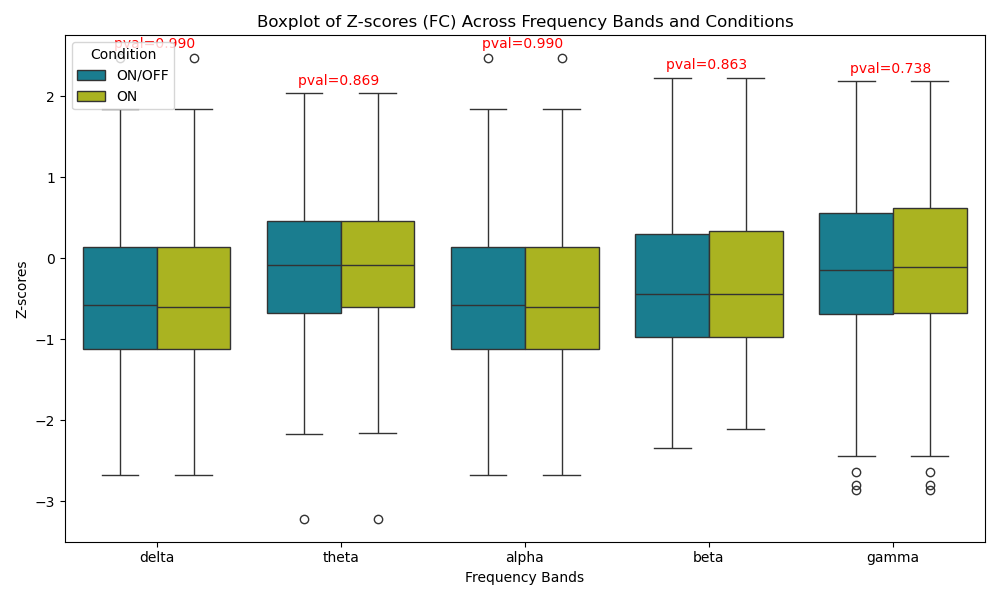
**

**Supplementary Figure 20 | Boxplots of z-score deviations for FC features across frequency bands, comparing ON/OFF-medication and ON-only medication conditions for PD patients.** Mann-Whitney U test p-values are indicated in red.

**
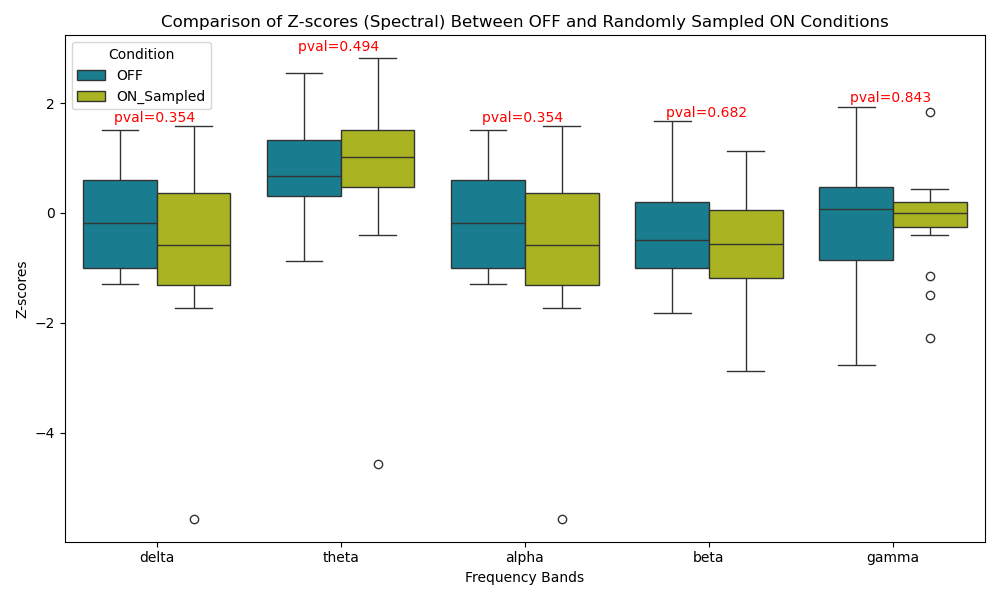
**

**Supplementary Figure 21 | Boxplots of z-score deviations for spectral features across frequency bands, comparing OFF-medication conditions with a randomly sampled subset of ON-medication conditions for PD patients.** Mann-Whitney U test p-values are indicated in red.

**
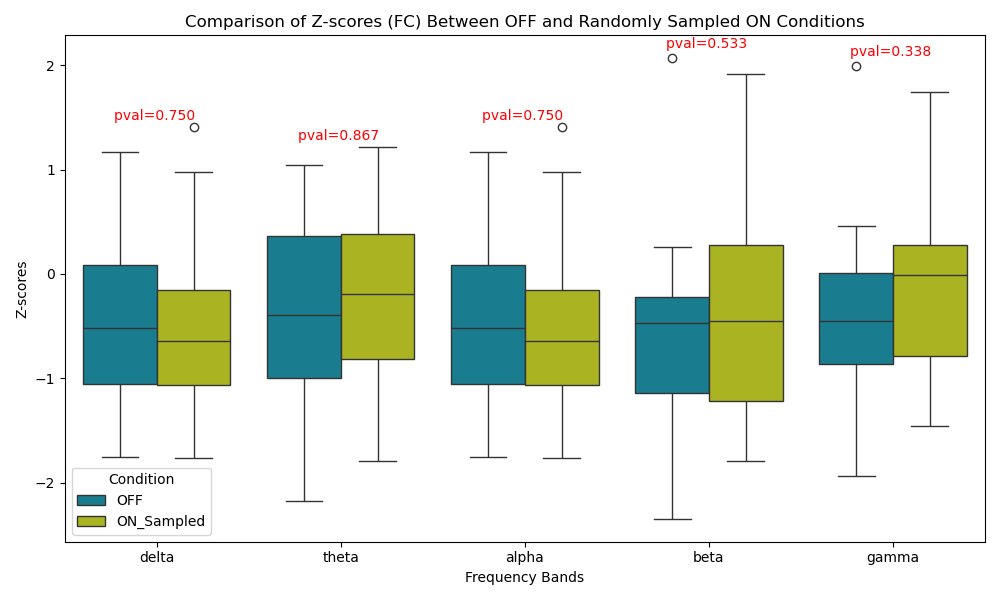
**

**Supplementary Figure 22 | Boxplots of z-score deviations for FC features across frequency bands, comparing OFF-medication conditions with a randomly sampled subset of ON-medication conditions for PD patients.** Mann-Whitney U test p-values are indicated in red.

## **References**

1. Miltiadous, A. *et al.* A Dataset of Scalp EEG Recordings of Alzheimer’s Disease, Frontotemporal Dementia and Healthy Subjects from Routine EEG. *Data* **8**, 95 (2023).

2. May, E. S. *et al.* Dynamics of brain function in patients with chronic pain assessed by microstate analysis of resting-state electroencephalography. *Pain* **162**, 2894–2908 (2021).

3. Yassine, S. *et al.* Functional Brain Dysconnectivity in Parkinson’s Disease: A 5‐Year Longitudinal Study. *Mov Disord* **37**, 1444–1453 (2022).

4. Cavanagh, J. F., Kumar, P., Mueller, A. A., Richardson, S. P. & Mueen, A. Diminished EEG habituation to novel events effectively classifies Parkinson’s patients. *Clin Neurophysiol* **129**, 409–418 (2018).

5. Chhade, F. *et al.* Predicting creative behavior using resting-state electroencephalography. *Commun Biol* **7**, 1–11 (2024).

6. Güntekin, B. *et al.* Impairment in recognition of emotional facial expressions in Alzheimer’s disease is represented by EEG theta and alpha responses. *Psychophysiology* **56**, e13434 (2019).

7. Zanin, M. *et al.* Reconstructing brain functional networks through identifiability and deep learning. *Network Neuroscience* **8**, 241–259 (2024).

8. Kabbara, A. *et al.* Reduced integration and improved segregation of functional brain networks in Alzheimer’s disease. *J. Neural Eng.* **15**, 026023 (2018).

9. Allouch, S. *et al.* Altered motor performance in Alzheimer’s disease: a dynamic analysis using EEG. in *2019 Fifth International Conference on Advances in Biomedical Engineering (ICABME)* 1–4 (IEEE, Tripoli, Lebanon, 2019). doi:10.1109/ICABME47164.2019.8940204.

10. Paban, V., Deshayes, C., Ferrer, M.-H., Weill, A. & Alescio-Lautier, B. Resting Brain Functional Networks and Trait Coping. *Brain Connectivity* **8**, 475–486 (2018).

11. Railo, H. *et al.* Resting state EEG as a biomarker of Parkinson’s disease: Influence of measurement conditions. 2020.05.08.084343 Preprint at https://doi.org/10.1101/2020.05.08.084343 (2020).

12. Schalk, G., McFarland, D. J., Hinterberger, T., Birbaumer, N. & Wolpaw, J. R. BCI2000: a general-purpose brain-computer interface (BCI) system. *IEEE Transactions on Biomedical Engineering* **51**, 1034–1043 (2004).

13. Hatlestad-Hall, C. *et al.* Reliable evaluation of functional connectivity and graph theory measures in source-level EEG: How many electrodes are enough? *Clin Neurophysiol* **150**, 1–16 (2023).

14. Fuglsang, S. A., Märcher-Rørsted, J., Dau, T. & Hjortkjær, J. Effects of Sensorineural Hearing Loss on Cortical Synchronization to Competing Speech during Selective Attention. *J Neurosci* **40**, 2562–2572 (2020).

15. American Psychiatric Association. *Diagnostic and Statistical Manual of Mental Disorders: DSM-5*. vol. 5 (2013).

16. McKhann, G. M. *et al.* The diagnosis of dementia due to Alzheimer’s disease: recommendations from the National Institute on Aging-Alzheimer’s Association workgroups on diagnostic guidelines for Alzheimer’s disease. *Alzheimers Dement* **7**, 263–269 (2011).

17. Lang, A. E. & Lozano, A. M. Parkinson’s disease. First of two parts. *N Engl J Med* **339**, 1044–1053 (1998).

18. Berg, L. Clinical Dementia Rating (CDR). *Psychopharmacol Bull* **24**, 637–639 (1988).

19. Postuma, R. B. *et al.* MDS clinical diagnostic criteria for Parkinson’s disease. *Mov Disord* **30**, 1591–1601 (2015).

20. Daniel, S. E. & Lees, A. J. Parkinson’s Disease Society Brain Bank, London: overview and research. *J Neural Transm Suppl* **39**, 165–172 (1993).

21. Hoehn, M. M. & Yahr, M. D. Parkinsonism: onset, progression and mortality. *Neurology* **17**, 427–442 (1967).

22. Cockrell, J. R. & Folstein, M. F. Mini-Mental State Examination (MMSE). *Psychopharmacol Bull* **24**, 689–692 (1988).
